# Role of laser ablation synthesis parameters in ORR electrocatalytic performance of MOF-derived hybrid nanocomposites†

**DOI:** 10.1039/d5ra04056f

**Published:** 2025-07-18

**Authors:** Mahshid Mokhtarnejad, Erick L. Ribeiro, Soheil Almasi, Bamin Khomami

**Affiliations:** a Department of Chemical & Biomolecular Engineering, University of Tennessee Knoxville Tennessee 37996 USA bkhomami@utk.edu; b Material Research and Innovation Laboratory (MRAIL), University of Tennessee Knoxville Tennessee 37996 USA; c Department of Mechanical Engineering, Polytechnic School of the University of São Paulo São Paulo SP 05508-030 Brazil erickribeiro@usp.br; d Research Centre for Greenhouse Gas Innovation (RCGI), University of São Paulo São Paulo SP 05508-030 Brazil

## Abstract

This study presents a rapid, eco-friendly, and scalable method for fabricating non-precious metal electrocatalysts for the oxygen reduction reaction (ORR) using Laser Ablation Synthesis in Solution (LASiS). We demonstrate that by optimizing the laser output power and ablation time, Co-based metal–organic frameworks (MOFs) can be directly synthesized and converted into hybrid nanocomposites composed of Co_3_O_4_, CoO, and metallic Co embedded in nitrogen-doped carbon. These materials exhibit high porosity, stable crystalline structures, and enhanced ORR activity, including a four-electron transfer pathway, excellent durability, and performance comparable to commercial Pt/C catalysts. Compared with traditional hydrothermal methods, LASiS provides a template-free and solvent-minimizing alternative that enables precise control over particle size, structure, and composition in a single-step process. This work highlights the potential of LASiS as a powerful tool to develop next-generation sustainable energy materials.

## Abstract 2

This study presents a rapid, eco-friendly, and scalable method for fabricating non-precious metal electrocatalysts for the oxygen reduction reaction (ORR) using Laser Ablation Synthesis in Solution (LASiS).

## Graphical abstract

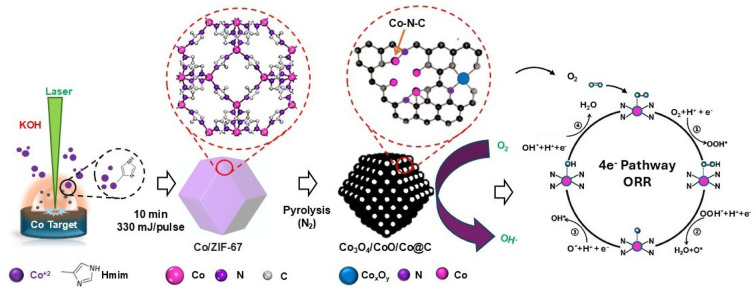

## Introduction

Electrochemical energy conversion technologies, particularly fuel cells, have become essential components of the sustainable energy infrastructure because of their high efficiency and low environmental footprint. Various types of fuel cells have been developed to date, including polymer electrolyte membrane fuel cells (PEMFC),^1^ anion exchange membrane fuel cells (AEMFC),^2^ alkaline fuel cells,^3^ solid oxide fuel cells (SOFCs),^4^ and molten carbonate fuel cells,^5^ to meet different energy demands. Among the different types of fuel cell technologies, AEMFCs offer notable advantages such as reduced reliance on expensive platinum group metal (PGM) catalysts and extended operational life in alkaline environments. Despite such advantages, the performance of AEMFC systems is hindered by the sluggish kinetics of the oxygen reduction reaction (ORR) at the cathode, which involves a complex four-electron transfer process and requires efficient and cost-effective electrocatalysts to accelerate the reaction kinetics.^6^

To address the limitations of ORR in AEMFCs, significant research has focused on developing high-performance catalysts that can replace conventional platinum-based nanoparticles (NPs). Although platinum-based catalysts exhibit excellent activity, their high cost and poor stability in alkaline environments have led to the search for alternatives, nonprecious metal-based electro-catalysts.^7^ In this regard, carbon-based hybrid nanocomposites derived from a metal–organic framework (MOF), particularly zeolitic imidazolate frameworks (ZIF), have emerged as promising candidates because of their tunable porosity, high surface area, superior electron conductivity, and ability to host active Co–N–C sites.^8–11^ Upon pyrolysis, ZIFs, specifically Co/ZIF-67, transform into nitrogen-doped carbon structures embedded with cobalt or cobalt oxide phases, which are recognized for their ORR activity.^12^ However, optimizing the size, crystallinity, structure, and morphology of these catalysts is essential to maximize the number of accessible active sites and to improve mass transport during electrochemical reactions.^13,14^ Unfortunately, conventional methods for MOF synthesizing often suffer from long reaction times and limited control over particle size and morphology.^15^ Furthermore, residual solvents and unreacted precursors can compromise material purity and environmental sustainability.^16^ Therefore, rapid, green, and morphology-controllable synthesis techniques are needed to fully realize the electrocatalytic potential of MOF-derived hybrid nanocomposites, where precise structural tuning is needed for electrocatalytic applications.

In this study, we employ the Laser Ablation Synthesis in Solution (LASiS) technique as a rapid, cost-effective, and environmentally friendly method to synthesize a Co MOF, named ZIF-67, with controlled morphology and crystallinity. By tuning key LASiS parameters such as laser output power, ablation time, and postpyrolysis conditions, we aim to precisely control the size, crystallinity, and morphology of these highly porous particles as templates to enhance the electrochemical performance of the MOF-derived HNCs. After pyrolysis, the resulting Co_3_O_4_/CoO/Co@C HNCs are investigated as nonprecious metal electrocatalysts for ORR, offering a cost-effective alternative to Pt and other PGM catalysts. The optimal ZIF-67 platform was synthesized with a 10 minutes ablation time and an output power of 330 mJ per pulse, demonstrating the highest electrocatalytic performance after post-treatment at 500 °C. Detailed structural and electrochemical characterizations were used to reveal the correlation between synthesis conditions, nucleation dynamics, and active Co–N–C sites, ultimately providing mechanistic insights into the increased ORR performance of these catalysts. This work highlights the significance of LASiS parameters during MOF synthesis as a scalable pathway for designing high-performance, non-PGM electrocatalysts.

## Experiments and methods

The methodology involves the synthesis, structural control, and optimization of ZIF-67 and its derived HNCs as ORR electrocatalysts, achieved by tuning the laser parameters during the LASiS process to tailor their size and morphology.

### ZIF-67-based HNCs synthesized *via* optimized laser ablation synthesis in solution (LASiS)

In this study, we synthesized Co-based metal–organic frameworks (Co/ZIF-67) using laser ablation synthesis in solution (LASiS). LASiS involves the laser ablation of a Co-metal target submerged in a liquid medium, forming a laser-induced plasma plume at the solid–liquid interface. In this work, we refer to the product as Co/ZIF-67 to distinguish it from conventionally synthesized ZIF-67, since cobalt ions are introduced by laser ablation of a metallic cobalt pellet rather than from a dissolved salt precursor. This approach enables precise control over composition and purity. The elevated temperature and pressure of the laser-derived plasma facilitate unique chemical reactions, enabling the synthesis of novel material phases by combining elements from the liquid and the metal target. Upon cooling and depressurization, the plasma allows for cluster growth and the formation of nanoparticles (NPs) and nanocrystals. In the context of MOF synthesis, although conventional methodologies, such as hydrothermal and solvothermal approaches, are widely employed, the synthesis of ZIF-67 through these routes presents several notable limitations, including high energy consumption, prolonged reaction times, stringent pressure and temperature requirements, limited scalability, broad particle size distributions, labor-intensive post-synthetic treatments, reliance on hazardous or costly solvents, and environmental concerns associated with solvent waste and inherent inefficiencies of batch processing.^17^ In contrast, LASiS offers a rapid, environmentally benign, and template-free alternative, allowing precise control over particle size and morphology, positioning itself as a particularly attractive strategy for the scalable and sustainable production of ZIF-67 and derivatives. For more details on the LASiS technique, see the following publications.^18–22^

**Fig. 1:**
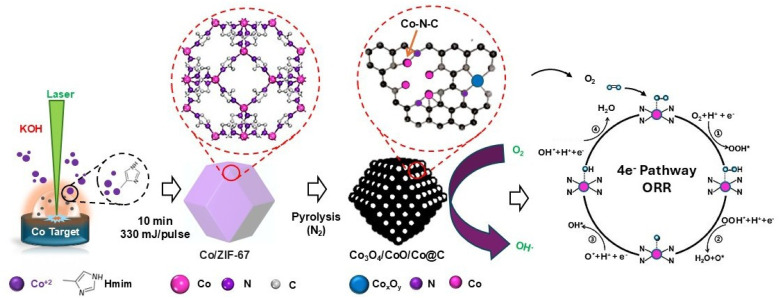
Schematic illustration of LASiS methodology used to synthesize Co/ZIF-67-derived ORR electrocatalysts.

For the synthesis of Co/ZIF-67, we used an unfocused high-energy pulsed Nd:YAG laser (1064 nm) to ablate a cobalt target (Co) immersed and immobilized in an alkaline solution. We used potassium hydroxide (KOH, >99%) to adjust the solution to a pH of 13.3. Then, we added 1.8 g of 2-methylimidazole (Hmim) to 40 mL of the alkaline solution containing dissolved KOH (3 M) in DI water. A high hydroxyl concentration, provided by initial addition of KOH, is essential, as Hmim deprotonation is not feasible at lower pH values due to its high p*K*_a_ (p*K*_a_(Hmim) = 14.2).^23^ All the required precursors and materials were obtained from Sigma-Aldrich and cobalt (Co) pallets with 1¼ inch diameter, 1¼ inch height and 99.5% purity, obtained from Kurt J. Lesker. A 1064 nm pulsed Nd-YAG laser with a 10 Hz repetition rate is employed for LASiS. To control the ZIF structures and optimize the laser parameters, ablation was performed at two different laser output powers (110 mJ per pulse and 330 mJ per pulse) for durations of 5, 10, 15, and 20 minutes. To facilitate the crystallization of Co/ZIF-67, given the inherently slow nucleation and crystallization kinetics of MOFs even in alkaline media, the suspensions were allowed to rest at room temperature for a period of 24 hours. Subsequently, the laser-synthesized products were centrifuged at 4700 rpm for 15 minutes, followed by sequential washing with deionized water and methanol multiple times to thoroughly eliminate any residual unreacted precursors. The final product was then treated with a pyrolysis process at various temperatures (500 to 800 °C) under N_2_ conditions for 2 hours to obtain HNCs based on ZIF Co_3_O_4_/CoO/Co@C with optimized structure and morphology for optimal ORR electrocatalyst performance. The LASiS process is schematically illustrated in Fig. 1.

#### Structural, compositional, and morphological characterization of MOFs and HNCs

Several analytical methods were applied to characterize the synthesized samples structurally and compositionally. XRD analysis was performed using a Phillips X'Pert-Pro diffractometer equipped with a Cu Kα monochromatic radiation source (*λ* = 0.1541 nm) operated at 45 kV and 40 mA. Fourier transform infrared (FTIR) spectroscopy was performed using a Nicolet iS50 spectrometer in an absorption range of 5000–400 cm^−1^. Surface morphology and elemental composition were assessed by scanning electron microscopy (SEM) using a Philips XL-30E microscope equipped with energy-dispersive X-ray (EDX) spectroscopy, along with a Helios 5UX dual-beam focused ion beam SEM (FIB/SEM) system for acquiring SEM-EDS images. Furthermore, transmission electron microscopy (TEM) was performed using both a JEOL JEM 1400-Flash microscope and a Thermofisher Tecnai Osiris scanning/transmission electron microscope (S/TEM), both operating at 120 kV accelerating voltage.

Raman spectroscopy was performed using an NT-MDT NTEGRA spectroscopy apparatus, employing a 532 nm green laser excitation. Thermogravimetric analysis (TGA) was carried out on a TA Instruments Q50 15 GA analyzer under a nitrogen (N_2_) atmosphere, heating samples from room temperature to 700 °C at a rate of 10 °C min^−1^. These analyzes were essential to determine the optimal pyrolysis conditions and thermal behavior of the laser-synthesized Co/ZIF-67 and Co_3_O_4_/CoO/Co hybrid nanocomposites (HNC). Overall, the characterization results confirmed the controlled synthesis and full crystallization of ZIF-based and hybrid structures suitable for electrocatalytic applications.

#### Electrochemical characterization

Electrochemical testing was conducted using rotating disk electrode (RDE) setups from Pine Instruments LLC. The cell featured a three-compartment design, incorporating a saturated double-junction Ag/AgCl electrode as the reference electrode and a platinum coil as the counter electrode. All electrochemical potentials measured were converted and reported relative to the reversible hydrogen electrode (RHE) scale.

#### Oxygen reduction reaction (ORR) tests

Although most reports on ORR electrocatalysts in alkaline media typically rely on RDE measurements conducted in solutions of 0.1 M alkali hydroxide solutions, this study employs a more concentrated electrolyte (1 M KOH) to deliberately impose harsher testing conditions. This elevated hydroxide concentration serves to critically probe the structural and electrochemical stability of the LASiS-derived NCs under aggressive alkaline environments representative of real-world ORR operations. Beyond accelerating degradation mechanisms, high-alkalinity electrolytes introduce additional complexity to ORR catalysis due to reduced oxygen solubility and diffusivity, as well as increased surface blockage from adsorbed hydroxyl species.^18^ Therefore, performance evaluation under such stringent conditions provides a more rigorous and realistic measure of the catalytic robustness and functional viability of the synthesized NCs. For comparison of catalytic activity and stability, we selected a commercially sourced electrocatalyst composed of 20 wt% platinum supported on BASF SE carbon as a reference. The catalyst inks were made by mixing 2 mg of the prepared HNCs with 25 μL of a 5 wt% Nafion solution in 1 mL of ethanol. Then we applied 6 μL of this ink on the RDE surface, achieving a catalyst loading of approximately 30 μg cm^−2^. The electrocatalytic behavior was characterized by performing rotating disk voltammetry (RDV) measurements at different rotation rates, and data analysis was carried out using the Koutecky–Levich equation (KL) (eqn (1)).1
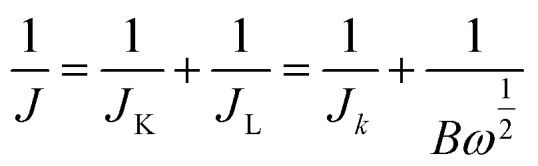
*J*_K_ = *nFkC*_0_; *B* = 0.62*nFC*_0_*D*_0_^2/3^*ν*^−1/6^, where *J* is the total current density, *J*_K_ is the kinetic limiting current density, and *J*_L_ is the diffusion limiting current density; *n* depicts the number of transferred electrons, *F* denotes the Faraday constant, *C*_0_ is dissolved O_2_ concentration, and *D*_0_ is the O_2_ diffusion coefficient in the electrolyte; *ν* represents the rotational rate of the electrode in revolution per minute (rpm). The kinetic currents *J*_K_ for all Tafel plots were derived from eqn (2):2
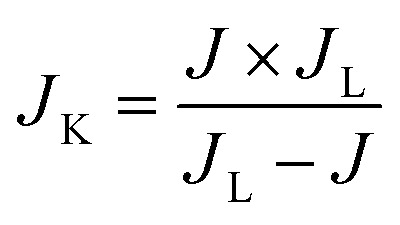


## Results and discussion

### Optimization of the LASiS parameters to control the size and structure of Co/ZIF-67

**Fig. 2:**
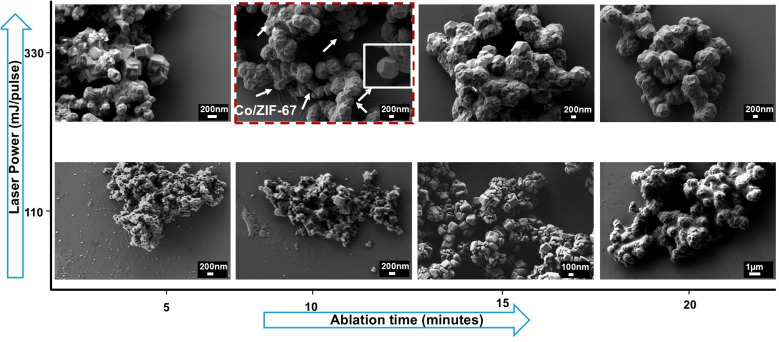
SEM micrographs of Co/ZIF-67 nanostructures synthesized using LASiS technique at laser output powers of 110 and 330 mJ per pulse, and ablation times ranging from 5 to 20 minutes.

As mentioned earlier, the LASiS process was carried out by ablating a Co pellet immersed in KOH and Hmim solution for durations ranging from 5 to 20 minutes using two different laser output powers: 330 mJ per pulse and 110 mJ per pulse. The laser ablation time and output power play critical roles in determining the structure and morphology of the synthesized Co/ZIF-67 nanomaterials, as shown in Fig. 2. At a high laser output power of 330 mJ per pulse, a short ablation time of 5 minutes is insufficient for the complete formation of the MOF, resulting in small particles that exhibit cubic structures. Increasing the ablation time to 10 minutes leads to well-defined, fully grown hexagonal structures, indicating optimal MOF formation. Extending the ablation time to 15 and 20 minutes leads to significant agglomeration in the ZIF morphology, leading to loss of optimal structure and potentially reducing the final functionality of these MOFs. However, with a lower laser output power of 110 mJ per pulse, the system lacks enough energy to generate adequate concentrations of Co^2+^ cations through the ablation of the metal target, leading to slow nucleation processes with the formation of defective structures. Although a 15 minutes ablation at this lower power produces smaller particles that begin to grow, extending the ablation time to 20 minutes results in severely aggregated structures with compromised morphology and potential functionality. These findings demonstrate the importance of carefully tuning laser output power and ablation time to achieve highly crystalline Co/ZIF-67 nanomaterials. SEM micrographs of Fig. 2 clearly show the morphological evolution and aggregation tendencies under varying synthesis conditions. Unlike conventional methods such as hydrothermal synthesis, which are time-consuming and solvent-intensive, LASiS offers a rapid, green, and template-free approach with precise control over particle size and morphology, making it especially attractive for scalable and sustainable nanomaterial production. In the context of MOF formation *via* LASiS, our previous studies^18,23^ have elucidated a comprehensive mechanistic framework detailing the reaction pathways initiated by high-energy laser ablation that culminate in the assembly of metal–organic structures. Briefly, the laser ablation of a cobalt target in an aqueous medium generates a transient plasma plume characterized by extreme temperatures and pressures, leading to the formation of cobalt nanoparticles (NPs). These NPs undergo rapid oxidation to Co^2+^ ions driven by redox interactions with solution phase species. Subsequently, the Co^2+^ ions engage in a sequence of reactions with the organic linker 2-methylimidazole (Hmim), involving coordination, deprotonation, and oligomerization steps, which collectively facilitate the formation of the ZIF-67 framework.

**Fig. 3:**
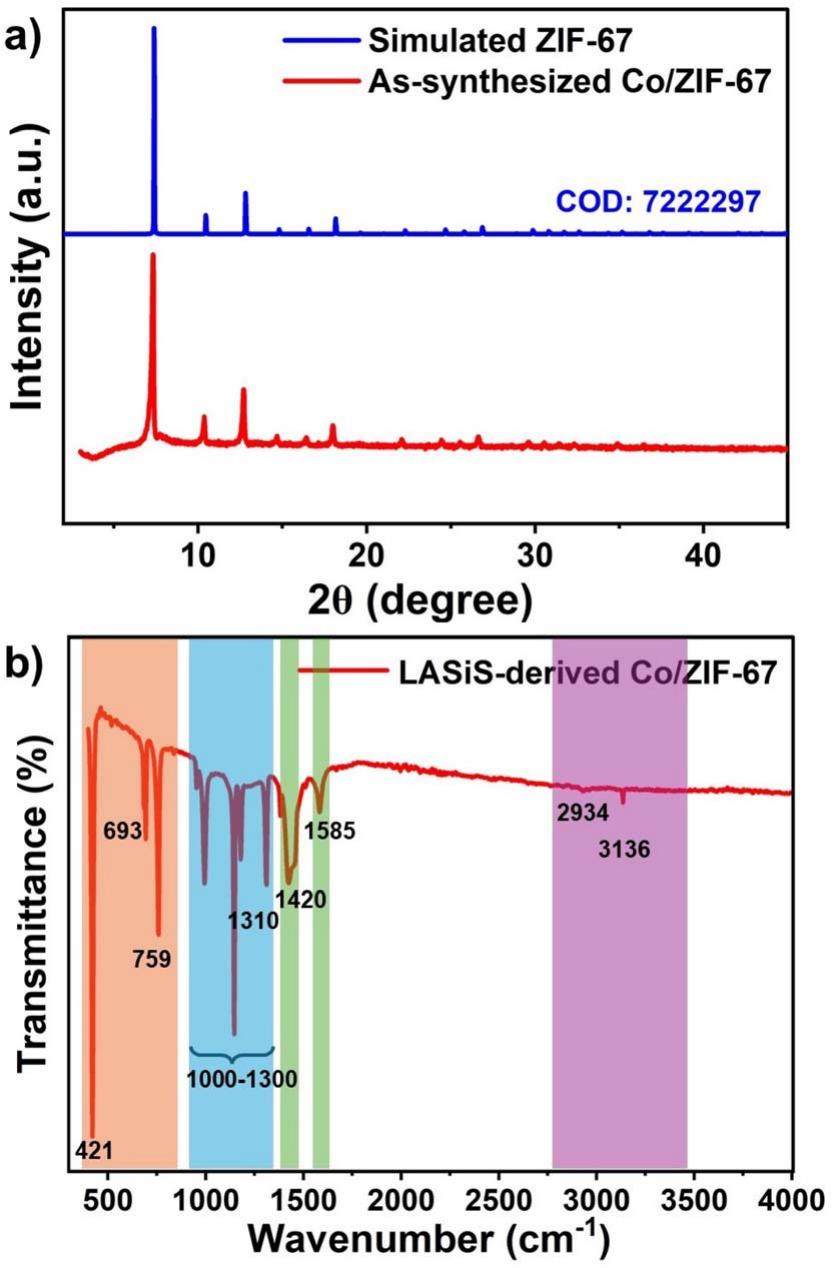
(a) XRD and (b) FTIR spectra of Co/ZIF-67 synthesized *via* LASiS under optimized conditions of 10 minutes ablation time and 330 mJ per pulse.

For structural characterization, XRD and FTIR analysis were performed on the Co/ZIF-67 sample obtained under optimal synthesis conditions (10 minutes ablation time at 330 mJ per pulse). XRD analysis shows the crystalline structure of Co/ZIF-67 by measuring the diffraction patterns produced when X-rays interact with the atomic planes in a crystal lattice. XRD provides information on the crystallinity, phase composition, and lattice parameters of synthesized materials.^24^ XRD patterns (Fig. 3(a)) of Co/ZIF-67 synthesized under optimized LASiS conditions (330 mJ per pulse, 10 min) show sharp and well-defined peaks matching the simulated ZIF-67, confirming phase purity and crystallinity of the MOFs. Complementing the XRD results, FTIR spectroscopy (Fig. 3(b)) was employed to identify key functional groups and verify the bonding environment within the synthesized MOF. FTIR is used to identify the functional groups and chemical bonds present in a material by analyzing the absorption of infrared light at various wavelengths, providing valuable information on molecular vibrations and bonding environments.^25^ Characteristic peaks observed in FTIR spectra (Fig. 3(b)), particularly those associated with Co–N stretching and imidazole ring vibrations, confirm the successful coordination between cobalt centers and organic ligands, supporting the formation of a well-defined ZIF-67 structure under LASiS conditions. In this case, the FTIR plot displays molecular vibrations characteristic of the 2-methylimidazole (Hmim) linker and metal–ligand coordination. The band at 759 cm^−1^ corresponds to the out-of-plane bending of the Hmim ring, while the peak at 1420 cm^−1^ is attributed to its in-plane bending vibrations.^26^ The prominent absorption bands at 1310 cm^−1^ and 1585 cm^−1^ correspond to stretching vibrations of C–N and C=N, respectively, which confirms the presence of Hmim as organic linker in the ZIF-67 framework.^27,28^ Additional peaks between 1300 and 1000 cm^−1^ further indicate a C–N stretching, whereas bands within the 1600 to 1500 cm^−1^ range are associated with vibrations of NH bending of the Hmim molecule. A weak peak at 1666 cm^−1^ arises from the stretching of the Hmim aromatic ring. In particular, the strong peak observed at 421 cm^−1^ is indicative of metal–nitrogen bonds (in this case, Co–N),^29^ validating the successful incorporation of metal centers into the ZIF structure. XRD and FTIR characterizations in Fig. 3(a) and (b) confirm the successful synthesis of structurally robust and highly crystalline Co/ZIF-67 frameworks under optimized LASiS conditions.

#### LASiS-derived Co_3_O_4_/CoO/Co@C HNCs as ORR catalyst

**Fig. 4:**
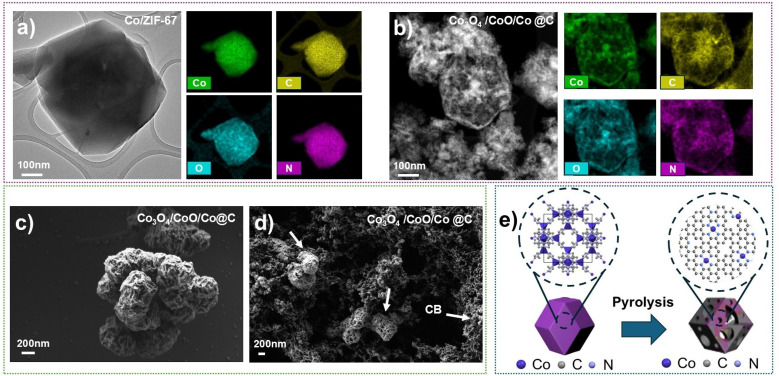
(a) Elemental mapping of Co/ZIF-67 before pyrolysis, (b) SEM micrographs of Co_3_O_4_/CoO/Co@C HNCs after pyrolysis at 500 °C under N_2_, (c) SEM micrograph of pyrolyzed sample before, and (d) after carbon black addition, (e) schematic illustration of the final Co/ZIF-67 and Co_3_O_4_/CoO/Co@C catalyst structure after pyrolysis.

After optimizing the laser parameters and controlling the morphology, the LASiS-derived Co/ZIF-67 requires a pyrolysis process to transform into Co_3_O_4_/CoO/Co@C HNC. This enhances conductivity, stability, and catalytic performance by forming a carbon-rich matrix with embedded cobalt-based active sites. Fig. 4 illustrates the structural and compositional transformations of Co/ZIF-67-derived materials for electrocatalytic applications. The initial elemental mapping of Co/ZIF-67 before pyrolysis (Fig. 4(a)) confirms the homogeneous distribution of Co, nitrogen (N), oxygen (O), and carbon (C), and the uniform incorporation of the metal centers within the MOF structure and the presence of 2-methylimidazole (Hmim) as a linker. By subjecting Co/ZIF-67 to pyrolysis at 500 °C under an inert N_2_ atmosphere, structural changes occur, as seen in Fig. 4(b) and S1.† Pyrolysis at 500 °C offers the optimal balance between carbon retention and crystallinity, preserving a porous, defect-rich carbon matrix with highly dispersed Co species that enhance ORR performance and long-term stability. As shown in the TGA data (Fig. S2†) and LSV curves (Fig. S3†), this temperature avoids overgrowth and excessive crystallization seen at ≥700 °C, resulting in superior catalytic activity and electron transport. This high temperature calcination process decomposes organic ligands and promotes the transformation of the MOF into a carbon-rich matrix with embedded cobalt oxide nanoparticles, yielding HNC denoted Co_3_O_4_/CoO/Co@C. Pyrolysis here serves a dual purpose: enhances the electrical conductivity of the material by converting the organic framework into conductive carbon and enables the formation of active metal sites (Co) crucial for electrocatalytic reactions. This carbonaceous matrix preserves the hierarchical nanostructure of the precursor MOF, providing a high surface area and improved electron transport pathways, which are essential for efficient ORR catalysis.

**Fig. 5:**
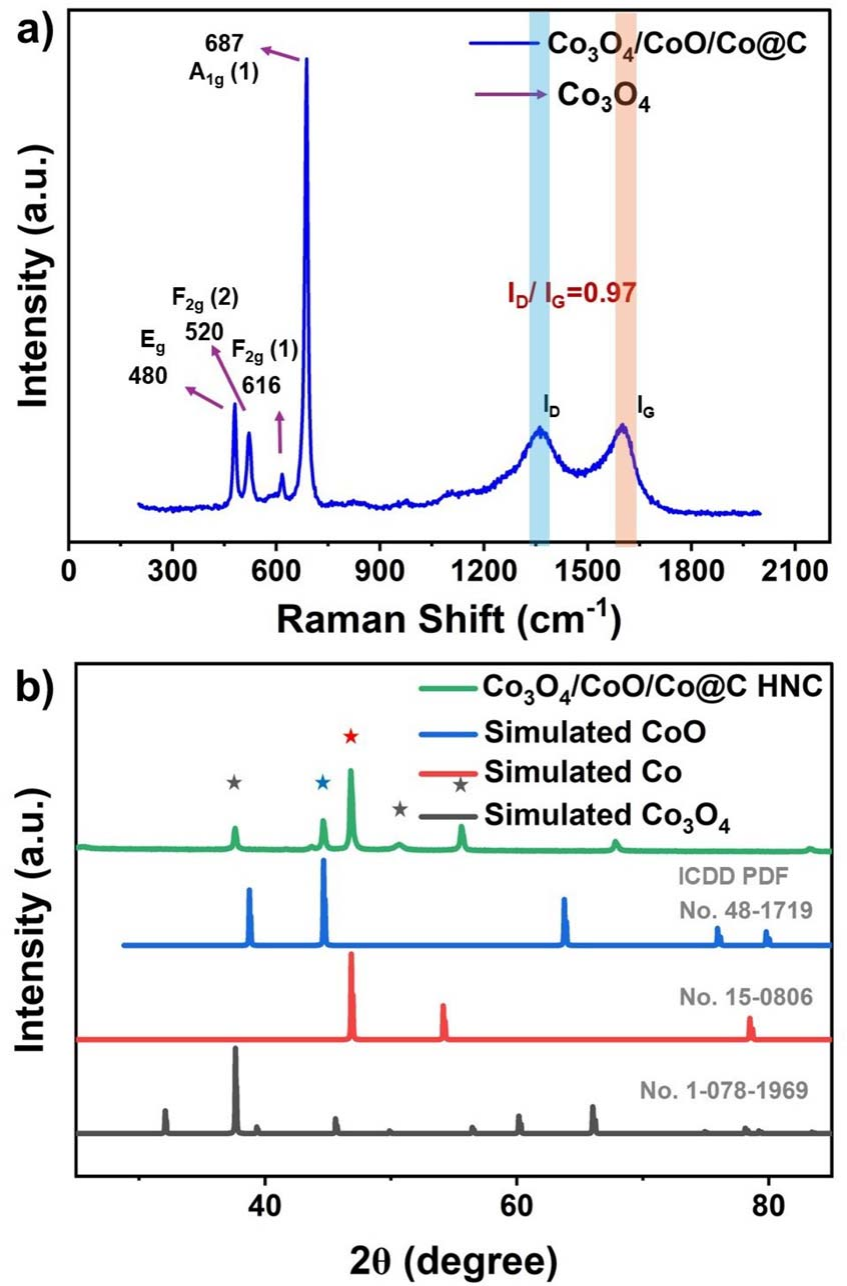
(a) Raman spectrum and (b) XRD pattern of Co_3_O_4_/CoO/Co@C nanocomposites.

To use these HNCs as ORR electrocatalysts, after the ZIF material undergoes calcination, the crystallinity, stability, and catalytic activity are further improved by enhancing the bonding between cobalt centers and the surrounding nitrogen-doped carbon (N–C) matrix. The strong interaction between Co nodes and the N-doped carbon framework forms Co–N–C active sites with a unique electronic configuration: the wavefunction of sp^2^ hybridized carbon networks overlaps with the electrons in the orbital d of the Co centers, producing a repulsion that upshifts the Fermi level of the system, resulting in a lower work function and consequently a more favorable charge transfer during the electrocatalytic process. In addition to that, excess valence electrons from N centers increase the local electron density of C–Co systems, leading to a further decrease in the work function of active catalytic sites.^18,20^ For practical application in electrochemical testing, calcined Co_3_O_4_/CoO/Co@C HNCs are dispersed into an ink solution containing carbon black (CB) and ethanol as the solvent, which improves the overall conductivity and improves the dispersion of the catalyst on the electrode surface. The SEM micrographs in Fig. 5(c) and (d) show the HNCs before and after the addition of carbon black. Before CB was added, the pyrolyzed MOF exhibits a somewhat collapsed, saggy carbon structure with visible Co nodes interconnected with nitrogen and carbon. After CB addition, mixing, and rigorous sonication of the electrocatalyst ink material, the carbonaceous material displays improved porosity and particle dispersion, which are critical for facilitating the access of the reactant to active sites during ORR. A schematic illustration in Fig. 4(e) shows the structural arrangement of the final catalyst, showing the Co centers coordinated to nitrogen and carbon within the conductive carbon framework. This well-engineered architecture ensures a good balance between high surface area, efficient electron transport, and abundant active sites, which, together, should enhance ORR activity and durability. This enhancement in ORR performance can be attributed to the hierarchical nanostructure, high surface area, and synergistic interactions between the Co_3_O_4_/CoO phases and the nitrogen-doped carbon matrix – observations that are consistent with recent studies on pyrolyzed MOF-derived systems.^30^

However, XRD patterns of the pyrolyzed Co/ZIF-67 samples showed sharp and well-defined peaks corresponding to Co_3_O_4_, CoO and metallic Co, indicating the formation of a multiphase crystalline structure. Raman spectra (Fig. 5(a)) further confirm the dominant Co_3_O_4_ spinel phase and show distinct D and G bands, suggesting the presence of graphitized carbon. The coexistence of Co_3_O_4_, CoO and metallic Co within a nitrogen-doped carbon matrix, as revealed by Raman and XRD analysis, points to a chemically complex yet stable hybrid network. These phases should originate from thermally driven transformations of Co–N coordination environments during pyrolysis, resulting in electronically and catalytically favorable structures. Post pyrolysis strong vibrational modes A_1g_ and F_2g_ are also observed in Fig. 5(b). The D and G bands (1350 and 1580 cm^−1^) reflect the presence of disordered and graphitic carbon, respectively.^31^Fig. 5(a) also highlights the carbon phases and the presence of Co metal oxides. The D band arises from the vibration mode A_1g_ associated with phonon symmetry, which is indicative of defects and disorder in the carbon structure. In contrast, the G band is attributed to the vibration E_2g_ in the plane of the sp^2^ hybridized carbon atoms, signifying the presence of graphitic carbon. The intensity ratio of these peaks (*I*_D_/*I*_G_), also shown in Fig. 5(a), serves as a useful metric to evaluate the degree of disorder *versus* graphitization within the carbon matrix. A higher *I*_D_/*I*_G_ ratio suggests an increased level of defects or amorphous carbon content, whereas a lower ratio indicates a more ordered graphitic structure. An *I*_D_/*I*_G_ ratio of 0.95 indicates a relatively balanced presence of both defective (amorphous) and graphitic (ordered sp^2^) carbon phases. In fact, although the electronic conductivity is highly desirable for the HNCs, the presence of dangling bonds arising from defects in the carbon matrix also plays a role in the electrocatalytic properties of the system. Although the mechanisms of these processes are not yet fully understood, other studies have reported that the reactive nature of unsatisfied C bonds can significantly contribute to the ORR electrocatalysis process.^32^

Taking this into account, the D/G ratio calculated from the Raman spectra ratio suggests that there is a moderate level of structural disorder, with significant graphitization still present,^33^ representing a desirable balance between an electronic conductor phase and the presence of active electrocatalyst C sites. In addition to the contribution peaks of the carbon D and G bands, the Raman spectra exhibit characteristic peaks corresponding to Co_3_O_4_, further confirming the incorporation of the metal oxide phases. The presence of well-defined graphitic carbon and Co_3_O_4_ indicates the formation of highly graphitic carbon–metal oxide composites. Thus, Co_3_O_4_/CoO/Co@C HNC possesses a well-developed graphitic structure while retaining enough defects to potentially enhance properties such as electrical conductivity and active sites for catalysis, which is especially beneficial when combined with Co_3_O_4_ in carbon–metal oxide composites and thus enhances the overall catalytic performance of HNCs. Therefore, the Raman spectrum of the pyrolyzed Co/ZIF-67 shows distinct D and G bands, confirming the formation of a carbonaceous matrix, along with characteristic peaks corresponding to Co_3_O_4_. The absence of CoO peaks, in contrast to the XRD data in Fig. 5(b), where both Co_3_O_4_ and CoO phases are present, can be attributed to the Raman-inactive nature of CoO due to its highly symmetric cubic structure.^34^ This suggests that Co_3_O_4_ forms Raman-active nodes embedded within a conductive carbon network, while CoO remains undetectable under Raman excitation.

Fig. 5(b) shows the XRD pattern of the material after calcination at 500 °C, which confirms the successful formation of hollow HNCs Co_3_O_4_/CoO/Co@C. The diffraction peaks agree excellently with the standard cubic phase of Co_3_O_4_/CoO/Co@C, with prominent reflections observed at 2*θ* values of approximately 19.0°, 31.3°, 36.8°, 44.8°, 59.3°, and 65.2°. These characteristic peaks verify the formation of highly crystalline CoO and Co_3_O_4_ following the calcination process. The successful incorporation of CoO and Co_3_O_4_ into the ZIF-67-derived carbon matrix results in a composite structure that benefits from the high catalytic activity of CoO and Co_3_O_4_ and the conductivity and structural stability provided by the carbon shell. This synergistic combination improves not only the stability and durability of the catalyst but also its overall electrochemical performance.

The existence of different phases of cobalt oxide may come from uniformly distributed cobalt ions within an organic framework during rapid energy input and localized heating during LASiS. Upon pyrolysis, the intense thermal decomposition of the organic ligands liberates gaseous byproducts and leaves behind a porous carbon matrix embedded with cobalt species. The cobalt ions, initially stabilized within the MOF architecture, undergo phase transformations depending on the local pyrolysis environment. Specifically, variations in temperature, oxygen partial pressure, and carbonization atmosphere promote the formation of different phases of cobalt oxide. In regions with limited oxygen availability and rapid thermal gradients, CoO tends to form as a result of the partial oxidation of cobalt. In contrast, areas with slightly higher oxygen diffusion or prolonged exposure to elevated temperatures favor the formation of Co_3_O_4_, a mixed valence compound. As a result, the pyrolysis of ZIF-67 nanostructures synthesized with LASiS leads to a hybrid material containing CoO and Co_3_O_4_ phases, which will enhance electrochemical performance by combining the properties of both oxides.^35,36^

### Structure-controlled ZIF-67 as ORR electrocatalyst

In this section, we present the ORR electrocatalytic performance and long-term stability of HNC derived from LASiS Co_3_O_4_/CoO/Co@C.

#### Electrochemical and functional characterization of Co_3_O_4_/CoO/Co@C HNCs

**Fig. 6:**
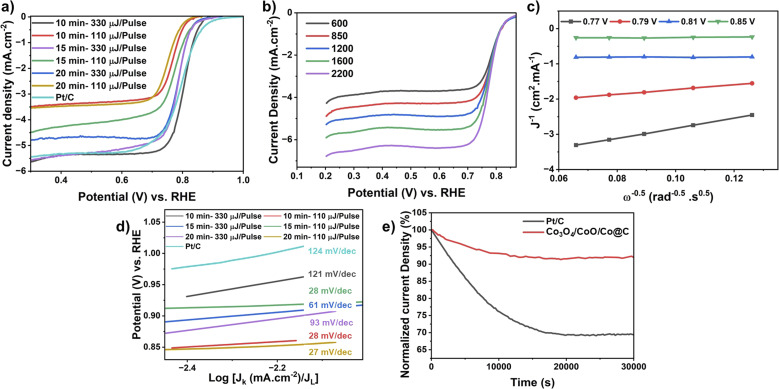
(a) Linear sweep voltammetry (LSV) curves of samples synthesized at varying ablation times and laser powers, (b) LSV curves of the optimized catalyst at different rotation speeds (600–2200 rpm), (c) corresponding Koutecky–Levich (K–L) plots, (d) Tafel plots of samples synthesized at different ablation times and laser powers, (e) chronoamperometric stability plots of Pt/C and optimized HNC.

A 5 mm diameter polished carbon RDE was employed as the working electrode for the cathodic and ORR electrocatalytic evaluations. 1 M KOH was used as the electrolyte. Commercial platinum supported on carbon (C/Pt, 20%, BASF) was used as a benchmark catalyst for comparison. While 0.1 M KOH is typically used as the standard electrolyte for ORR studies in alkaline media, the use of 1 M KOH introduces a more challenging environment, allowing for a more rigorous assessment of the performance and durability of the LASiS-derived hierarchical HNCs. However, operating in such a highly alkaline medium presents additional complexities. The lower solubility and diffusivity of oxygen, along with extensive surface coverage by alkaline species, make it more difficult to accurately assess the electrocatalytic behavior of the synthesized HNCs. As a result, evaluating their true ORR performance under these conditions becomes significantly more demanding. For this means, Fig. 6 provides electrochemical insights into ORR performance of Co_3_O_4_/CoO/Co@C hierarchical HNCs synthesized *via* LASiS under varying ablation time from 5 to 20 minutes and laser power from 110 to 330 mJ per pulse. Fig. 6(a) shows the linear sweep voltammetry (LSV) curves for HNCs derived from post-pyrolysis of LASiS-derived Co-ZIF-67 at different ablation times (10, 15, 20 minutes – samples prepared under 5 minutes ablation were not selected for the test due to very small quantity and agglomeration in the morphology) and laser energies (110 and 330 mJ per pulse). Notably, the sample synthesized at 10 minutes ablation time using a high-power laser exhibits the lowest ORR onset potential and the highest limiting current density, highlighting its superior catalytic activity. These findings correlate well with structural characterizations (SEM graphs and XRD plots), which confirm the formation of highly crystalline and porous MOF frameworks under these optimized synthesis conditions. The favorable morphology and surface structure formed under high-energy, 10 minutes ablation time likely facilitate better electron transport and reactant accessibility, crucial for high-performance ORR catalysis, which is remarkably very close to C/Pt. Fig. 6(b) illustrates the LSV curves at different rotation speeds (600 to 2200 rpm) for the optimal HNC sample (10 min, high laser power and low laser Q-switch). The clear increase in current density with rotation speed indicates a diffusion-controlled ORR process. The associated Koutecky–Levich (K–L) plots in Fig. 6(c) exhibit linearity with parallel slopes, suggesting a first-order reaction kinetics with respect to dissolved O_2_ and confirming a four-electron transfer pathway. This is critical for maximizing energy conversion efficiency and minimizing the formation of undesirable peroxide intermediates. Fig. 6(d) presents the corresponding Tafel plots derived from LSV data in Fig. 6(a). The optimized HNC exhibits a low Tafel slope, indicative of favorable reaction kinetics and a fast ORR process. Compared to the other LASiS-derived samples, the optimized catalyst (10 min ablation time, high laser power) demonstrates the most efficient charge transfer, likely due to synergistic effects between the Co_3_O_4_ active sites, the porous framework, and the conductive carbon support. The controlled hexagonal morphology and nanoscale size of Co/ZIF-67 synthesized under optimal LASiS conditions directly contribute to enhanced ORR activity by promoting a higher density of accessible active sites, improved mass transport, and more efficient electron conductivity in the resulting pyrolyzed HNCs. Finally, Fig. 6(e) compares the chronoamperometric stability of the optimized Co_3_O_4_/CoO/Co@C HNC against a commercially used C/Pt catalyst in O_2_-saturated 1 M KOH solution. While the C/Pt catalyst shows a pronounced decline in current density over time, the HNC maintains remarkable stability, with only minimal performance loss even after extended operation. Notably, the Co_3_O_4_/CoO/Co@C sample exhibited excellent long-term durability, retaining over 95% of its initial ORR current density even after 30 000 cycles. In contrast, the commercially used C/Pt catalyst showed a significantly greater performance degradation, with its current density decreasing by approximately more than 30% under the same testing conditions. This emphasizes the robust electrochemical durability of LASiS-synthesized Co_3_O_4_/CoO/Co@C HNCs, making them promising candidates as ORR electrocatalysts. The presence of catalytically active Co–N–C moieties and interconnected cobalt oxide domains in the HNCs, as evidenced by XRD and FTIR analyses, can be directly correlated with the improved ORR electrocatalytic performance observed in the electrochemical measurements. Nitrogen doping in carbon-based materials, particularly in conjunction with cobalt, plays a pivotal role in modulating the electronic structure and enhancing ORR activity. Nitrogen functions as an electron donor, increasing the local electron density and shifting the Fermi level toward the conduction band, thus improving the charge transfer across the carbon matrix.^37^ In Co–N–C systems, nitrogen incorporation not only promotes efficient electron transport but also contributes to the formation of catalytically active sites by stabilizing cobalt species, either as atomically dispersed centers or as nanoparticles.^38^ This synergistic interaction between nitrogen and cobalt facilitates the adsorption and activation of O_2_, thus accelerating the overall reduction kinetics and accounting for the high catalytic performance. These results support the statement that a 10 minutes ablation under high-power laser pulses (330 mJ per pulse) results in the most effective HNC morphology and electrochemical performance. These outcomes stress the influence of LASiS parameters on structural and functional characteristics of MOF-based electrocatalysts and support the importance of tuning synthesis conditions during LASiS for optimal catalytic behavior.

## Conclusion

This study demonstrates the successful application of LASiS in the engineering of Co-based HNCs derived from MOFs as high-performance nonprecious metal electrocatalysts for ORR. By precisely tuning the laser output power and ablation time, we achieved fine control over the size and crystallinity of the Co/ZIF-67 precursors, resulting in pyrolyzed HNCs composed of a mixed phase Co_3_O_4_/CoO/Co embedded in a conductive carbon matrix, where CoO dominates the Raman response due to its stable crystalline structure, while CoO remains undetected due to its symmetric cubic phase, despite both being visible in XRD. The optimized LASiS conditions led to highly porous crystalline structures that exhibited a four-electron ORR pathway, excellent durability, and performance comparable to those of commercial Pt/C catalysts. Compared with pyrolyzed hydrothermal MOFs, LASiS-derived hybrid nanocomposites offer superior structural purity, optimized nanoscale morphology, and enhanced ORR activity with excellent durability, demonstrating the potential of this scalable method for next-generation electrocatalyst developments.

## Author contributions

MM and BM conceived the experiments, MM conducted the experiments, and MM, SA, ER, and BB analyzed the results. MM, ER, and BK contributed to the writing of the manuscript.

## Conflicts of interest

There are no conflicts to declare.

## Supplementary Material

RA-015-D5RA04056F-s001

## Data Availability

The datasets generated and/or analyzed during the current study are not publicly available due to ongoing data protection and confidentiality considerations, but are available from the corresponding author upon reasonable request. The data can be made available to reviewers for the purpose of peer review.
