# Supplementary material for: Role of laser ablation synthesis parameters in ORR electrocatalytic performance of MOF-derived hybrid nanocomposites
Source: RSC Adv. 2025 Jul 18;15(31):25707–16. doi: 10.1039/d5ra04056f (PMC12273548; doi:10.1039/d5ra04056f)
Supplement: RA-015-D5RA04056F-s001 [file RA-015-D5RA04056F-s001.pdf]

Supplementary Information (SI) for RSC Advances.
This journal is © The Royal Society of Chemistry 2025

Supplementary Information (ESI)

Role of Laser Ablation Synthesis Parameters in ORR Electrocatalytic Performance of MOF-derived Hybrid Nanocomposites

Mahshid Mokhtarnejad,[a,b] Soheil Almasi,[a,b], Erick L. Ribeiro,[c] and *Bamin Khomami[a,b]

a Department of Chemical & Biomolecular Engineering, University of Tennessee,
Knoxville, Tennessee, 37996, USA.

b Material Research and Innovation Laboratory (MRAIL), University of Tennessee,
Knoxville, Tennessee, 37996, USA.

c Department of Mechanical Engineering, Polytechnic School of the University of São
Paulo, São Paulo, 05508-030, SP, Brazil.

* Corresponding Author: bkhomami@utk.edu

This supplementary information supports our manuscript by providing additional insights into the development of Co/ZIF-67-derived HNCs synthesized via LASiS. We aim to demonstrate how varying pyrolysis temperatures influence the catalytic performance of Co/ZIF-67-based materials. Through SEM and TEM imaging, we showed the coexistence of cobalt oxide particles and the presence of randomly dispersed metal domains. Additionally, TGA analysis reveals the decomposition behavior of the precursor and justifies the optimal post-treatment temperature window (500 - 700 °C) based on carbon retention and structural evolution. These findings provide some background for understanding the enhanced electrocatalytic performance observed under specific synthesis conditions.

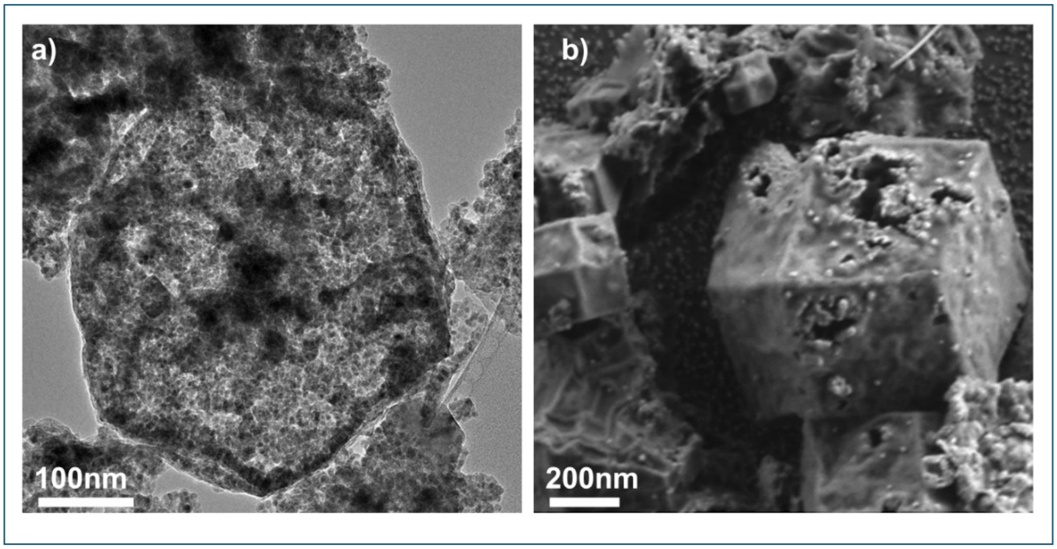

Fig S 1. a) TEM and b) SEM images of the Co/ZIF-67-derived HNCs after pyrolysis at 500 °C. The SEM images reveal cobalt oxide morphologies, including cubic, hexagonal, and irregular

granular shapes. These structural differences suggest the coexistence of mixed cobalt oxide phases such as CoO and Co_3O_4 within the porous carbon matrix. The structural heterogeneity is attributed to rapid decomposition of the MOF framework during pyrolysis and localized temperature / oxygen variations within the carbon network.

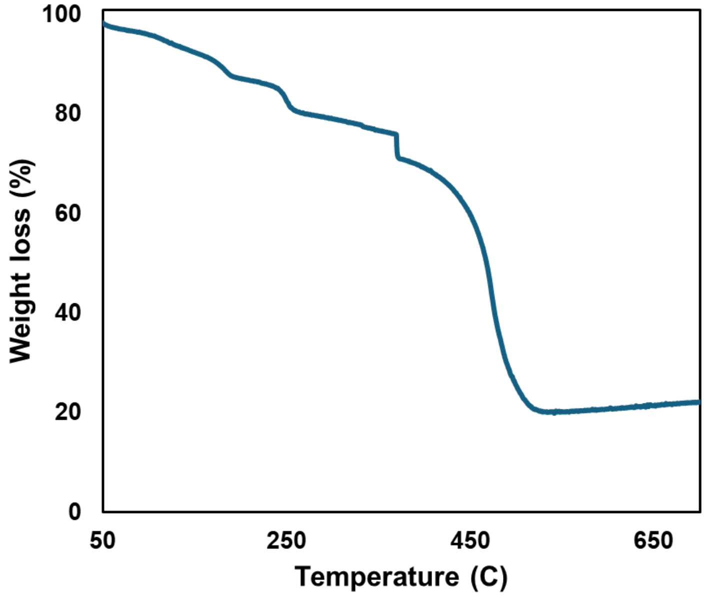

Fig S 2.TGA was conducted to understand the thermal decomposition behavior of the Co/ZIF-67 precursor: Below 200 °C, mass loss corresponds to evaporation of water and solvents. Around 300 °C, major decomposition of the imidazole ligand occurs. At 700 °C, approximately 20% of the initial mass remains, indicating the presence of carbonaceous residue and cobalt oxides. This residual mass is essential to form the conductive carbon framework and cobalt oxide phases necessary for ORR catalysis. The data supports the chosen pyrolysis temperature range of 500 - 700 °C.

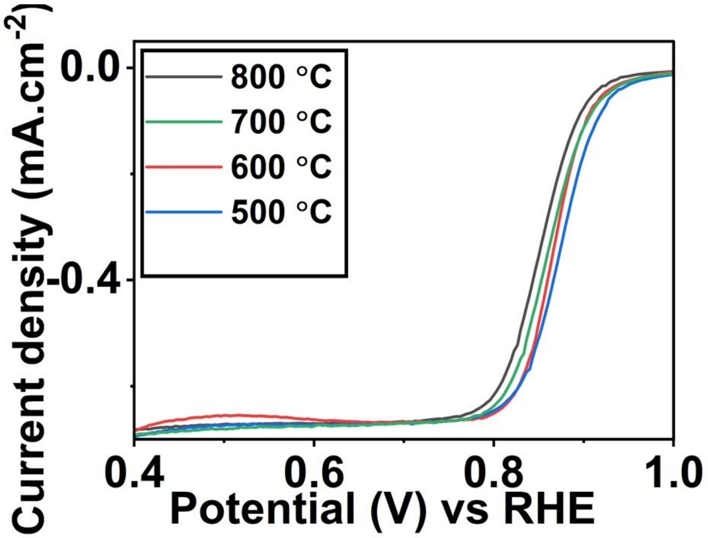

Fig S 3. While higher pyrolysis temperatures increase crystallinity and conductivity, 500 °C strikes a balance by retaining sufficient carbon content, avoiding particle overgrowth, and enabling a

defect-rich, porous matrix with highly dispersed Co species, leading to enhanced ORR catalytic performance and long-term stability. At higher temperatures (≥700 °C), the Co particles may become overly crystalline or fuse into larger grains, which reduces the number of accessible active sites. Pyrolysis at 500 °C preserves small, catalytically active domains and avoids over-crystallization. Although TGA analysis shows that ~20% of the original mass remains at 700 °C, but at 500 °C more carbon may be retained. This leads to a more robust conductive carbon network, improving electron transfer during electrocatalysis. Also, at 500 °C, the MOF-derived carbon framework retains more of its original porosity and fine structure, which helps maintain a high surface area - crucial for mass transport during ORR.
